# Audiological Outcomes of Weekly vs. Triweekly Cisplatin in Head and Neck Cancer with Cochlear-Sparing Intensity-Modulated Radiation Therapy

**DOI:** 10.3390/cancers16122228

**Published:** 2024-06-14

**Authors:** Mauricio E. Gamez, Dukagjin M. Blakaj, Priyanka Bhateja, Amy Custer, Brett G. Klamer, Jeff Pan, Emile Gogineni, Sujith Baliga, Marcelo R. Bonomi

**Affiliations:** 1 Department of Radiation Oncology, Mayo Clinic Rochester, Rochester, MN 55905, USA; gamezharo.mauricio@mayo.edu; 2 Department of Radiation Oncology, The Ohio State University Wexner Medical Center, Columbus, OH 43210, USA; dukagjin.blakaj@osumc.edu (D.M.B.); emile.gogineni@osumc.edu (E.G.); sujith.baliga@osumc.edu (S.B.); 3 Department of Medical Oncology, The Ohio State University Wexner Medical Center, Columbus, OH 43210, USA; priyanka.bhateja@osumc.edu; 4 Oncology Rehabilitation Team, The Ohio State University Wexner Medical Center, Columbus, OH 43210, USA; amy.custer@osumc.edu; 5 Center for Biostatistics, Department of Biomedical Informatics, College of Medicine, The Ohio State University, Columbus, OH 43210, USA; brett.klamer@osumc.edu (B.G.K.); jeff.pan@osumc.edu (J.P.)

**Keywords:** ototoxicity, audiological outcomes, weekly cisplatin, high-dose cisplatin, head and neck cancer, IMRT, adverse events

## Abstract

**Simple Summary:**

Cisplatin-based chemoradiation is the standard of care for patients with squamous cell carcinomas of the head and neck area. Recent data from randomized phase 3 studies showed that weekly cisplatin has the same oncological outcomes as high-dose triweekly cisplatin when combined with definitive radiation. In this study, audiologic data were prospectively collected before, during, and after treatment completion. Standard audiogram evaluation was used in all cases. The primary endpoint was a hearing change grade of ≥3 (CTCAE v5.0) after completion of chemoradiation. The high-dose cisplatin regimen significantly increased the ≥grade 3 severe irreversible ototoxicity risk compared to the low-dose weekly regimen, irrespective of cumulative cisplatin dose, even with the use of cochlear-sparing intensity-modulated radiation therapy. No significant difference in oncologic outcomes was observed between the two schedules. These findings should be validated in a larger prospective multi-institutional study.

**Abstract:**

Cisplatin, one of the most ototoxic anti-neoplastic agents, causes permanent hearing loss in up to 90% of patients. We assessed ototoxicity rates and prospectively collected audiologic outcomes of patients receiving low-dose or high-dose cisplatin with concurrent cochlear-sparing intensity-modulated radiation therapy (IMRT). Patients with head and neck squamous cell carcinoma (HNSCC) receiving definitive or adjuvant cisplatin-based chemoradiotherapy (CRT) were analyzed. Cisplatin was administered either in low doses weekly (40 mg/m^2^) for up to seven doses or in high doses triweekly (100 mg/m^2^) for up to three doses. Cochlear-sparing IMRT was delivered in all cases. Audiologic data were prospectively collected before, during, and after treatment completion. The primary endpoint was a hearing change grade of ≥3 after CRT completion. Of the 96 HNSCC patients evaluated, 69 received weekly cisplatin and 58 received definitive CRT. Of patients receiving weekly cisplatin, 13% developed ≥G3 ototoxicity vs. 56% of patients who received triweekly cisplatin (*p* < 0.001). In multivariable modeling, the cisplatin dose schedule remained significant (OR: 8.4, 95%CI: 2.8–27.8, *p* < 0.001) for risk of severe irreversible ototoxicity. Triweekly cisplatin CRT significantly increased the ≥G3 severe irreversible ototoxicity risk compared to low-dose weekly cisplatin, irrespective of the cumulative cisplatin dose, even with the use of cochlear-sparing IMRT. No significant difference in oncologic outcomes was observed between the two schedules.

## 1. Introduction

The management of locally advanced head and neck squamous cell carcinoma (HNSCC) consists of multimodal therapy, with either definitive concurrent chemoradiotherapy (CRT) or surgery followed by adjuvant chemoradiotherapy based on high-risk pathologic features. High-dose triweekly (100 mg/m^2^) cisplatin remains the standard chemotherapy agent for the treatment of HNSCC [1,2,3,4,5,6]. While progress has been made in the last two decades to improve oncologic outcomes, treatment-related toxicities continue to significantly impact the long-term function and quality of life of patients with head and neck cancer [7,8]. Triweekly cisplatin has been shown to be associated with significant toxicities such as acute kidney injury, neutropenia, dehydration, and hearing loss [9]. Recently, several groups have transitioned to the use of low-dose weekly cisplatin (20–50 mg/m^2^) due to better tolerability and patient compliance [10,11,12]. In addition, two recent randomized trials showed that the use of weekly 40 mg/m^2^ cisplatin is superior to cetuximab in terms of cancer control [13] and is not inferior to triweekly cisplatin in the adjuvant setting for patients with high-risk oral cavity cancers [14]. However, the optimal weekly and total cumulative dose and whether this scheme has comparable oncologic outcomes and lower toxicity rates compared to standard high-dose cisplatin remain an area of debate and ongoing investigation. 

Hematologic, gastrointestinal, neurologic, and ototoxic adverse effects related to cisplatin are well known [15]. Sensorineural hearing loss (SNHL) and tinnitus are particularly disabling adverse effects of cisplatin exposure and are reported in up to 90% of patients undergoing platinum-based CRT [16]. To date, several factors have been implicated in the risk of hearing loss, including patient-related factors (age, gender, baseline hearing function, nutritional status, albumin concentrations, environmental noise exposure), tumor-treatment-related factors (cisplatin schedule and total cumulative dose, radiation dose(s) to the cochlea(s), radiation fractionation and treated volume), and tumor location [17,18,19]. 

Similar to cisplatin-induced ototoxicity, the pathophysiology of radiation-induced SNHL is not completely understood, though vascular insufficiency and microscopic anatomic connective tissue changes in inner ear (cochlea) structures have been postulated as potential mechanisms for biologic damage. Additionally, there are limited dosimetric data with respect to radiation therapy (RT) constraint doses to the cochlea when delivered alone or in combination with ototoxic cisplatin and the successive hearing impact. At present, the Quantitative Analyses of Normal Tissue Effects in the Clinic (QUANTEC) group recommends mean doses to the cochlea ≤ 45 Gy [19], or more conservatively ≤ 35 Gy, for conventionally fractionated RT to minimize the risk of SNHL, yet no specific dose threshold to prevent it is known, nor are there clear monitoring guidelines for adult patients receiving head and neck RT [20]. Therefore, this study aims to retrospectively evaluate the incidence of severe irreversible grade 3 (G3) ototoxicity in patients with HNSCC receiving either low-dose weekly or high-dose triweekly cisplatin with concurrent cochlear-sparing intensity-modulated radiation therapy (IMRT). 

## 2. Materials and Methods

We performed a retrospective review of patients with locally advanced HNSCC treated with curative-intent treatment either with low-dose (40 mg/m^2^, maximum seven cycles) weekly or high-dose (100 mg/m^2^, maximum three cycles) triweekly cisplatin and concurrent cochlear-sparing IMRT. The inclusion criterion included non-metastatic HNSCC of all head and neck mucosal subsites, as long as there was no skull base invasion. Patients must have been deemed eligible for cisplatin-based chemotherapy. Patients treated with palliative intent radiotherapy or patients with a tumor extending near the cochlea were considered ineligible for the study. Patients with missing radiotherapy data were excluded from the study population. Patient demographic and baseline clinical characteristics were also collected and evaluated, including age, sex, primary tumor site, tumor stage, baseline creatinine, baseline hearing loss, baseline tinnitus, number of chemotherapy cycles, cisplatin cumulative dose, cochlear RT dose, change to carboplatin, and number of audiologic evaluations. 

Prospective baseline (pre-treatment), monitoring (during treatment), and follow-up (post-treatment) audiologic evaluations were obtained in each case as part of our head and neck department ototoxicity protocol. Collection of data occurred between October 2016 and February 2018. This study was approved by the internal institutional review board (IRB). 

### 2.1. Radiation Planning

Patients were staged in accordance with the American Joint Committee on Cancer Staging System 7th edition. Computed tomography (CT) or magnetic resonance imaging (MRI) of the head and neck, in addition to a CT or positron emission tomography/CT (PET/CT) of the chest, was routinely performed. All patients were treated with cochlear-sparing IMRT with simultaneous integrated boost (SIB) using a volumetric arc therapy (VMAT) technique. The goal was to deliver a bilateral mean cochlear RT dose below 15 Gy, but up to 20 Gy was accepted. Delivered radiation doses were 6600–7000 cGy for definitive cases and 6000–6600 cGy at 200 cGy per fraction in the post-operative setting at the discretion of the treating physician. Three-dimensional (3D) CT planning scans were fused with pre-treatment staging diagnostic scans. Fine cuts (≤2 mm) were obtained at the skull base (temporal bone), and bone window levels were used for better identification of the organ at risk. Gross tumor volumes (GTVs) were determined by clinical findings and staging scans. Clinical target volumes (CTVs) were created by adding a 5 mm margin to GTVs. Air, bone, and anatomical boundaries were cropped out to finalize the CTV. Planning target volumes (PTVs) were obtained by adding a 3–5 mm margin to the CTVs to account for setup uncertainties. Delineation of the cochlea(s) was performed by a head and neck dosimetrist using a bone window and MRI when available and verified by a head and neck radiation oncologist. In addition, a planning organ at risk volume (PRV) with a 3 mm margin was created to further reduce the dose and spare the cochlea(s). All head and neck contours (target volumes and organs at risk including cochlea) and radiation treatment plans were evaluated and approved during our weekly head and neck interdisciplinary board review. The daily setup accuracy was consistently monitored by the use of image-guided radiation therapy with acquisition of MV/kV films and/or ConeBeam CT.

### 2.2. Chemotherapy Details

The eligibility of each patient to receive systemic therapy was based on the initial clinical stage or high-risk pathologic features (positive margins and/or extracapsular extension). The decision to recommend either low-dose or high-dose cisplatin was based on several clinical factors including the ability to meet the following criteria: ECOG performance status ≤2, calculated creatinine clearance ≥ 60 mL/min, adequate organ function, and moderate or severe sensorineural hearing loss on baseline audiologic exam. Patients with a significant change in their hearing status during CRT were changed to carboplatin.

### 2.3. Ototoxicity Evaluation

Ototoxicity evaluations included otoscopy, tympanometry, ipsilateral acoustic reflex thresholds (500, 1000, 2000, and 4000 Hz), pure-tone air-conduction audiometry (250–8000 Hz), pure-tone bone-conduction audiometry (500–4000 Hz), high-frequency audiometry (9000–20,000 Hz), sensitive range for ototoxicity (SRO), speech reception threshold (SRT), word recognition score (WRS), and distortion product otoacoustic emission (OAE) (1500–10,000 Hz in 1/6th octave increments) assessments. Evaluations took place at baseline and then after administration of every other dose for individuals receiving weekly cisplatin and after each dose for individuals receiving triweekly cisplatin. In the event that a significant change in hearing was noted, the frequencies with changed thresholds were retested within 24 h (usually immediately), and bone-conduction audiometry was performed and SRTs and WRSs were determined. A significant change in hearing status was defined as per the American Speech Language Hearing Association (ASHA) as (1) a 10 dB decrease in threshold at any two adjacent test frequencies, (2) a 20 dB decrease in threshold at any one test frequency, or (3) a loss of response at any three consecutive test frequencies where responses were previously obtained. Follow-up evaluations occurred 3–6 months after treatment completion. National Cancer Institute Common Terminology Criteria for Adverse Events (CTCAE v5.0) categorize hearing changes into the following grades for adults enrolled in a monitoring program based on a 1, 2, 3, 4, 6, and 8 kHz audiogram (Supplementary Materials Table S1).

### 2.4. Study Endpoints

The primary endpoint of the analysis was to determine the incidence of severe irreversible hearing change of ≥G3 after completion of CRT using the CTCAE v5.0 grading system. Secondary endpoints included evaluation of oncologic outcomes (overall survival, OS; progression-free survival, PFS; local and locoregional control, LCR) between the two cisplatin schedules. 

### 2.5. Statistics

Descriptive statistics were used to summarize baseline demographic, clinical, and treatment variables. Differences between cisplatin dose schedules were summarized using Wilcoxon’s rank sum test or Pearson’s chi-squared test. Audio measurements were summarized as means (standard deviation [17]) and comparisons between cisplatin schedules using Welch’s two-sample *t*-test. Univariable logistic regression was used to estimate the association between ototoxicity (G3 SNHL post-treatment) and variables such as cisplatin schedule, sex, age, CRT modality, mean cochlea dose (Gy), and total cisplatin cumulative dose. Multivariable logistic regression was used to model the association between ototoxicity and cisplatin schedule, adjusting for age, mean cochlea dose (Gy), and total cisplatin cumulative dose.

OS was defined as the date of initial radiation treatment to the date of death and censored at the date of last follow-up for those still alive. PFS was defined as the date of initial radiation to the date of progression or death and censored at the date of last follow-up for those still alive without progression. The log-rank test and Kaplan–Meier estimate of survival with a 95% confidence interval (CI) were used to compare survival outcomes between cisplatin schedule groups. Time to first G3 SNHL event was modeled using a Fine–Gray competing risks regression model, where death without experiencing G3 SNHL was a competing event.

Complete case analysis was used for all summary statistics and reported *p*-values and CIs were unadjusted for multiplicity. Statistical analyses were performed with R version 4.3.1 using the survival (version 3.5-5), gtsummary (version 1.7.2), and ggplot2 (version 3.4.2) packages.

## 3. Results

### 3.1. Baseline Characteristics

A total of 96 patients with locally advanced HNSCC comprised our study cohort: 69 were treated with weekly cisplatin (72%) and 27 (28%) were treated with triweekly cisplatin. The patient demographics and characteristics comparing the two cisplatin groups are shown in Table 1. The median age of the cohort was 57 years (IQR: 51, 62), and patients in the triweekly cisplatin group were more likely to be younger (51 vs. 59 years, *p* < 0.001). The majority of patients in this cohort had oropharyngeal HPV-mediated SCC (n = 56, 58%) and 50% of patients had stage III/IV disease. The median cisplatin cumulative total doses were 240 mg/m^2^ (IQR: 200, 260) and 200 mg/m^2^ (IQR: 175, 300; *p* = 0.6), and the median numbers of chemotherapy cycles were six (IQR: 5, 7) and two (IQR: 2, 3) in the weekly and triweekly cisplatin groups, respectively. The median cochlear RT dose was similar between the weekly (14 Gy; IQR: 8, 18) and triweekly (13 Gy; IQR 10, 15; *p* = 0.6) groups. The overall cohort had a high percentage of baseline hearing loss (77%) and tinnitus (31%), which did not differ based on the cisplatin dose schedule. Patients in the triweekly cohort were more likely to require a change to carboplatin (48% vs. 12%, *p* < 0.001). 

### 3.2. Disease Outcomes

Median follow-up for OS was 7.7 months (IQR: 5.6, 11.9) and was not significantly different between the cisplatin groups (*p* = 0.6). There was no significant difference in OS or PFS between the weekly and triweekly cisplatin groups (*p* = 0.14 and *p* = 0.36, respectively). One-year OS probability was 0.92 (95% CI: 0.85, 0.99) for the weekly and 0.84 (95% CI: 0.71, 1) for the triweekly cisplatin group. 

### 3.3. Ototoxicity Evaluation

Table 2 summarizes the patient survival and ototoxicity outcomes based on the cisplatin schedule. After treatment completion, a total of 24 (n = 96, 25%) patients experienced severe irreversible G3 SNHL. Specifically, 56% (n = 15/27) of patients who received triweekly cisplatin vs. 13% (n = 9/69) of patients who received weekly cisplatin developed G3 ototoxicity (*p* < 0.001). The incidence of tinnitus was not statistically different between groups (*p* = 0.4). On univariable analysis, the odds of ototoxicity were found to be related to the cisplatin schedule (*p* < 0.001), though unrelated to sex (*p* = 0.9), age (*p* = 0.067), CRT modality (*p* = 0.5), mean cochlea dose (Gy) (*p* = 0.7), and total cisplatin cumulative dose (*p* = 0.6). Adjusting for age, mean cochlea dose (Gy), and total cisplatin cumulative dose, triweekly patients had 8.4 times the odds (OR 95% CI: 2.8, 27.8) of experiencing ototoxicity compared to weekly cisplatin schedule patients. 

Table 3 demonstrates CTCAE hearing loss scores between cisplatin and mean RT cochlear doses. Among patients who received a mean cochlear dose of ≤20 Gy, the rate of ≥G3 SNHL was higher among the triweekly compared to the weekly cisplatin schedule cohort (58% vs. 12%). We were unable to establish a relationship at higher doses due to the low number of patients who were treated with >20 Gy to the cochlea. 

Although 24 patients had G3 SNHL at the last audiologic evaluation, 5 patients (4 weekly and 1 triweekly) experienced G3 SNHL during the treatment course with recovery to <G3 before the end of treatment. The cumulative incidence of a first G3 SNHL event was higher among the triweekly compared to the weekly cohort (HR: 5.81, 95% CI: 2.4, 13.9). At 6 months, the triweekly cumulative incidence of first G3 SNHL was 59% (95% CI: 38%, 75%) and the weekly cohort was 14% (95% CI: 7.4%, 24%).

Table 4 demonstrates point decibel modifications by frequency (kHz) between cisplatin regimens, showing more pronounced changes with triweekly cisplatin at high frequencies. 

**Figure 1:**
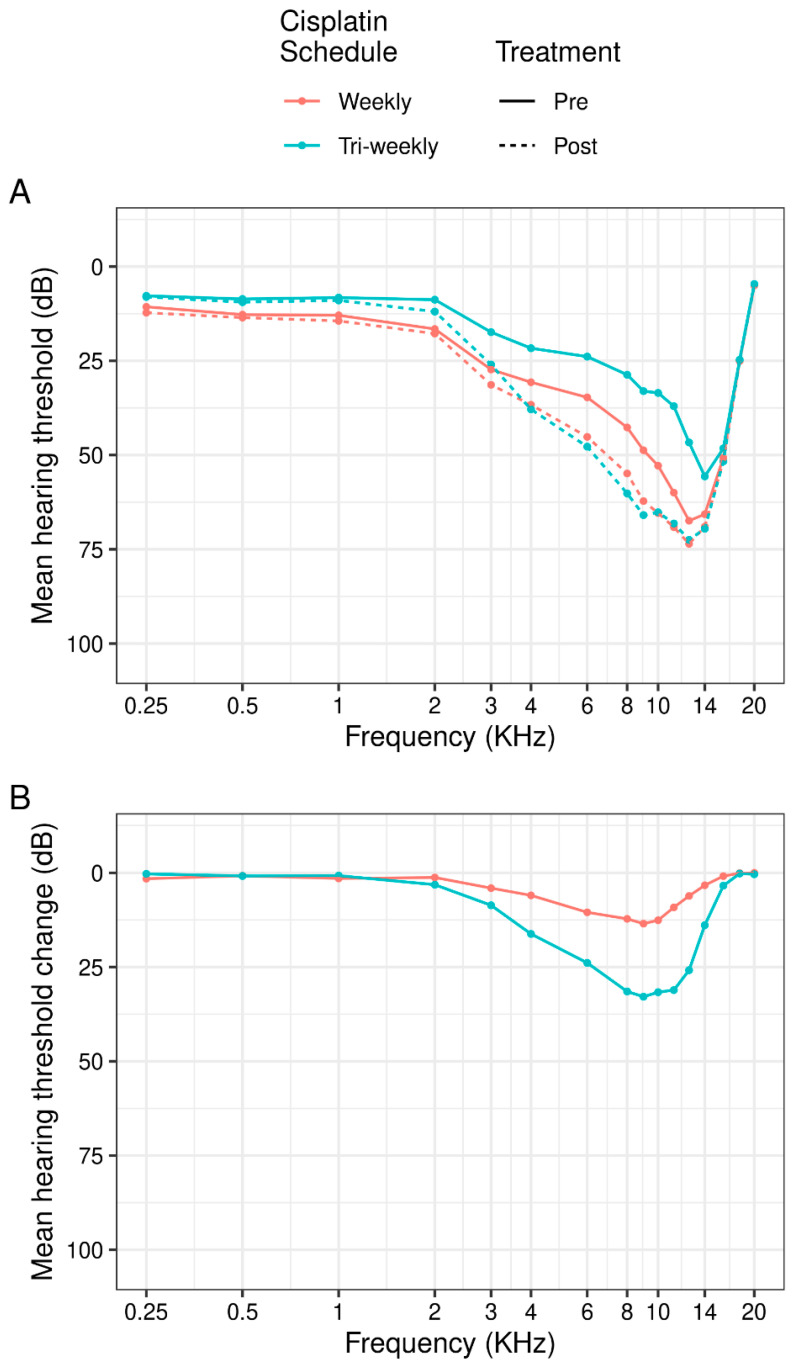
(**A**) Mean hearing threshold (dB) by cisplatin schedule and time of treatment and (**B**) the post-treatment change in mean hearing threshold (dB). Measurements averaged across left and right ears.

**Figure 2:**
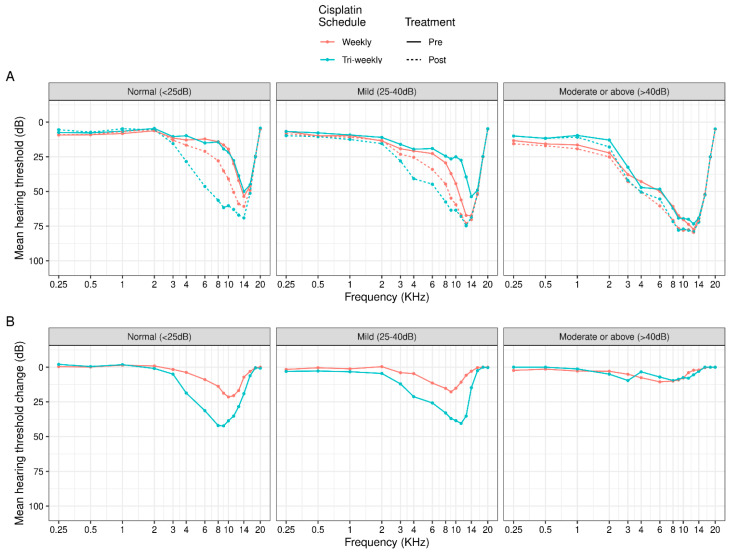
(**A**) Mean hearing threshold (dB) by cisplatin schedule and time of treatment stratified by baseline hearing loss and (**B**) the post-treatment change in mean hearing threshold (dB) stratified by baseline hearing loss. The normal hearing loss (<25 dB) group consisted of N = 14 weekly and N = 11 tri-weekly patients; the mild hearing loss (25–40 dB) group consisted of N = 19 weekly and N = 10 tri-weekly patients; and the moderate or above hearing loss (>40 dB) group consisted of N = 36 weekly and N = 6 tri-weekly patients. Measurements averaged across left and right ears.

Figure 1 shows the relationship between pre- and post-treatment audiologic measurements with respect to the cisplatin scheme, observing the significant changes in decibels at high frequencies for both cisplatin regimens, particularly in high-dose cisplatin patients. In addition, Figure 2 demonstrates the changes in decibels with frequency post-treatment in relation to baseline hearing, showing more notable differences in patients with a normal baseline hearing. 

## 4. Discussion

To date, no conclusive data have defined ototoxicity rates with the use of weekly versus triweekly cisplatin and concurrent cochlear-sparing IMRT for the management of patients with HNSCC. Here, we present a retrospective ototoxicity analysis, with our data suggesting significantly higher rates of severe irreversible ototoxicity with the use of triweekly cisplatin compared to weekly cisplatin, irrespective of the use of cochlear-sparing IMRT or the cisplatin total cumulative dose, and without a difference in oncologic outcomes.

The results from our study are congruent with those from other retrospective and prospective studies regarding the impact of triweekly cisplatin compared to weekly cisplatin on hearing. In the recently reported noninferiority ConCert trial, weekly cisplatin was found to be non-inferior to triweekly cisplatin in terms of locoregional control, but there were more toxicities in the triweekly arm [21]. In JCOG1008, patients with high-risk locally advanced SCC were randomized to triweekly cisplatin (100 mg/m^2^) vs. weekly cisplatin (40 mg/m^2^) and demonstrated non-inferiority of the weekly regimen. In addition, the rates of acute hearing disturbance and tinnitus were higher in the triweekly cisplatin arm [14]. Furthermore, a population-based study using Veterans Affairs (VA) data examined the outcomes of patients with locally advanced HNSCC treated with definitive intent CRT using either low-dose cisplatin (mean initial dose of 40 mg/m^2^) or high-dose cisplatin (mean initial dose of 100 mg/m^2^) and showed no difference in survival, yet high-dose cisplatin was associated with increased toxicity rates, including hearing loss [9]. In concordance with other previously published data, our results demonstrated no difference in oncologic outcomes between the two cisplatin groups, with post-treatment complete response of 86% and 78% (*p* = ns) for the patients receiving weekly 40 mg/m^2^ or triweekly 100 mg/m^2^ doses of cisplatin. 

One of the strengths of this study is that we evaluated hearing outcomes based on both the cisplatin schedule and cochlear RT dose. While the ototoxic effects of cisplatin and ionizing RT are well recognized, little is known about the incidence of severe irreversible hearing loss when comparing low-dose and high-dose cisplatin in combination with modern cochlear-sparing radiation therapy techniques. Prospective data from the University of Michigan showed an association between hearing loss at high frequencies (≥2000 Hz) and radiation dose to the cochlea, with changes in the bone conduction threshold of >10 dB more frequently occurring after mean cochlear doses ≥45 Gy [22]. Similarly, a systematic review of incidence rates of SNHL after RT or CRT in head and neck cancer showed an increased risk of hearing loss with doses >47 Gy [18]. Noteworthy is the large variability of the employed methods for hearing evaluation, the lack of adequate comparisons, the coalition of other confounding factors, and the scarcity of studies using modern radiation and normal organ-sparing RT techniques. Based on current data, QUANTEC recommends mean cochlear doses ≤45 Gy where possible while retaining the desired target coverage to minimize the risk of SNHL. Furthermore, even when these constraints are achieved, the risk is not negligible, with some reports showing up to 30% incidence of SNHL. Since a specific dose threshold cannot be safely determined, doses to the cochlea(s) should be kept as low as possible. Our data outline the importance of trying to further de-escalate doses to the cochlea with modern RT techniques when clinically indicated to assist with hearing preservation.

Several limitations must be acknowledged in our study. First, most patients in this study were treated with once-weekly cisplatin, limiting the sample size of the triweekly treated cohort (n = 27). In addition, this is a retrospective study and so is limited by selection bias. The follow-up was also short, so the long-term effect of cisplatin on ototoxicity is unclear. Next, only 11 patients were treated with a mean cochlear dose of >20 Gy, and these were mostly in the weekly cisplatin cohort. Therefore, we were unable to clearly determine the exact impact that higher doses of irradiation may have on the cochlea. Next, not all patients in our two cohorts completed the entire number of treatment cycles, with nearly 22% of triweekly cisplatin-treated patients only completing one cycle. Therefore, our ototoxicity rates may be underestimated. Finally, most of our measurements were objective and did not evaluate subjective self-perceived handicap of these ototoxicities using well-established questionnaires (The Hearing Handicap Inventory for Adults—Screening). 

## 5. Conclusions

In summary, our study has shown the risk of severe permanent (≥G3) ototoxicity by both the cisplatin dose and mean cochlear dose, which could have a bigger impact in the long-term function and quality of life of patients with head and neck cancer. After completion of CRT, 56% of patients who received a high dose of cisplatin developed ≥G3 SNHL in comparison with only 13% of those who received a low dose of cisplatin (*p* < 0.001, OR = 8.4). This risk was independent of age, gender, total cisplatin cumulative dose, primary tumor site, or CRT modality (definitive vs. adjuvant), and irrespective of the use of cochlear-sparing IMRT. Currently, NRG-HN009 is investigating whether once-weekly cisplatin is non-inferior to triweekly cisplatin in locally advanced HNSCC. In this study, mean cochlear doses of up to 35 Gy were considered acceptable. If the study meets the primary phase III objectives with regard to non-inferiority and toxicity, this may provide the opportunity for long-term HNSCC survivors to achieve a lower ototoxicity and preserve their quality of life. 

## Figures and Tables

**Table 1:** Patient demographics and clinical characteristics stratified by low-dose weekly vs. high-dose tri-weekly cisplatin schedules. Bold *p*-values denote those which have achieved statistical significance of *p* < 0.05.

Characteristic	Weekly, N = 69 ^1^	Tri-Weekly, N = 27 ^1^	*p*-Value ^2^	Overall, N = 96 ^1^
Age (years)	59 (53, 64)	51 (38, 58)	**<0.001**	57 (51, 62)
Sex			0.3	
Female	16 (23%)	3 (11%)		19 (20%)
Male	53 (77%)	24 (89%)		77 (80%)
Primary Site			0.8	
Oropharynx	38 (55%)	18 (67%)		56 (58%)
Larynx	11 (16%)	3 (11%)		14 (15%)
Oral Cavity	9 (13%)	4 (15%)		13 (14%)
Nasopharynx	4 (5.8%)	1 (3.7%)		5 (5.2%)
Unknown Primary	4 (5.8%)	0 (0%)		4 (4.2%)
Hypopharynx	2 (2.9%)	1 (3.7%)		3 (3.1%)
Para-nasal sinus	1 (1.4%)	0 (0%)		1 (1.0%)
Tumor Stage			0.2	
1	12 (17%)	1 (3.7%)		13 (14%)
2	21 (30%)	10 (37%)		31 (32%)
3	16 (23%)	6 (22%)		22 (23%)
4	16 (23%)	10 (37%)		26 (27%)
X	4 (5.8%)	0 (0%)		4 (4.2%)
Baseline Creatinine	0.80 (0.71, 0.95)	0.80 (0.68, 0.90)	0.7	0.80 (0.70, 0.94)
Baseline Hearing Loss			0.5	
No	14 (20%)	8 (30%)		22 (23%)
Yes	55 (80%)	19 (70%)		74 (77%)
Baseline Tinnitus			0.9	
No	47 (68%)	19 (70%)		66 (69%)
Yes	22 (32%)	8 (30%)		30 (31%)
Number of Chemotherapy Cycles				
1	0 (0%)	6 (22%)		6 (6.2%)
2	3 (4.3%)	11 (41%)		14 (15%)
3	1 (1.4%)	10 (37%)		11 (11%)
4	8 (12%)	0 (0%)		8 (8.3%)
5	19 (28%)	0 (0%)		19 (20%)
6	20 (29%)	0 (0%)		20 (21%)
7	18 (26%)	0 (0%)		18 (19%)
Cisplatin Cumulative Dose	240 (200, 260)	200 (175, 300)	0.6	210 (200, 280)
Change to Carboplatin			**<0.001**	
No	61 (88%)	14 (52%)		75 (78%)
Yes	8 (12%)	13 (48%)		21 (22%)
Cochlear RT dose (Gy)	14 (8, 18)	13 (10, 15)	0.6	13 (9, 17)
Number of Audiologic Evaluations				
2	4 (5.8%)	6 (22%)		10 (10%)
3	12 (17%)	11 (41%)		23 (24%)
4	36 (52%)	10 (37%)		46 (48%)
5	15 (22%)	0 (0%)		15 (16%)
6	2 (2.9%)	0 (0%)		2 (2.1%)

^1^ Median (IQR); N (%); ^2^ Wilcoxon rank sum test; Pearson’s Chi-squared test.

**Table 2:** Patient outcomes stratified by low-dose weekly vs. high-dose tri-weekly cisplatin schedules. Bold *p*-values denote those which have achieved statistical significance of *p* < 0.05.

Characteristic	Weekly, N = 69 ^1^	Tri-Weekly, N = 27 ^1^	*p*-Value ^2^	Overall, N = 96 ^1^
Disease Status			0.2	
Alive	64 (93%)	22 (81%)		86 (90%)
Death	5 (7.2%)	5 (19%)		10 (10%)
Hearing Loss on Follow Up			0.7	
No	6 (8.7%)	1 (3.7%)		7 (7.3%)
Yes	63 (91%)	26 (96%)		89 (93%)
Tinnitus on Follow Up			0.4	
No	23 (33%)	6 (22%)		29 (30%)
Yes	46 (67%)	21 (78%)		67 (70%)
Progressed Hearing Loss			0.4	
No	60 (87%)	21 (78%)		81 (84%)
Yes	9 (13%)	6 (22%)		15 (16%)
New or Worsening Tinnitus			0.3	
No	41 (59%)	12 (44%)		53 (55%)
Yes	28 (41%)	15 (56%)		43 (45%)
Last CTCAE Grade			**<0.001**	
0	36 (52%)	6 (22%)		42 (44%)
1	18 (26%)	2 (7.4%)		20 (21%)
2	6 (8.7%)	4 (15%)		10 (10%)
3	9 (13%)	15 (56%)		24 (25%)

^1^ N (%); ^2^ Pearson’s Chi-squared test.

**Table 3:** CTCAE hearing loss scores by cisplatin schedule and mean cochlear RT dose.

Cisplatin Schedule	Mean RT Cochlear Dose (Gy) ^1^	Grade 0, N = 42 ^2^	Grade 1, N = 20 ^2^	Grade 2, N = 10 ^2^	Grade 3, N = 24 ^2^
Weekly	0–20	32 (55%)	13 (23%)	6 (10%)	7 (12%)
	20–40	4 (40%)	4 (40%)	0 (0%)	2 (20%)
	40–70	0 (0%)	1 (100%)	0 (0%)	0 (0%)
Tri-weekly	0–20	6 (23%)	2 (7.7%)	3 (11%)	15 (58%)
	20–40	0 (0%)	0 (0%)	1 (100%)	0 (0%)
	40–70	0 (0%)	0 (0%)	0 (0%)	0 (0%)

^1^ Averaged across left and right ears. ^2^ N (%).

**Table 4:** Mean post-treatment change in decibels (post–pre) by frequency and cisplatin schedule.

Frequency (KHz)	Weekly, N = 69 ^1,2^	Tri-Weekly, N = 27 ^1,2^	*p*-Value ^3^
0.25	1.6 (6.4)	0.3 (4.9)	0.14
0.5	0.8 (6.0)	0.8 (4.9)	>0.9
1	1.5 (6.6)	0.7 (6.1)	0.5
2	1.2 (6.6)	3.1 (9.6)	0.2
3	4 (8)	9 (11)	**0.008**
4	6 (11)	16 (16)	**<0.001**
6	10 (14)	24 (20)	**<0.001**
8	12 (13)	31 (22)	**<0.001**

^1^ Averaged across left and right ears. ^2^ Mean (SD). ^3^ Welch’s two-sample *t*-test.

## Data Availability

Data for this analysis were made available through an IRB request and therefore will not be shared to protect patient anonymity.

## Supplementary Materials

The following supporting information can be downloaded at: https://www.mdpi.com/article/10.3390/cancers16122228/s1, Table S1: CTCAE v5.0 Hearing Changes.
