# Supplementary material for: Audiological Outcomes of Weekly vs. Triweekly Cisplatin in Head and Neck Cancer with Cochlear-Sparing Intensity-Modulated Radiation Therapy
Source: Cancers (Basel). 2024 Jun 14;16(12):2228. doi: 10.3390/cancers16122228 (PMC11201991; doi:10.3390/cancers16122228)
Supplement: Supplementary file 1 [file cancers-16-02228-s001.zip › cancers-3053227-supplementary.pdf]

Supplemental Table S1. CTCAE v5.0 Hearing Changes.

**Grade of Adverse Event**	**Definition**
**1**	Threshold shift of 15-25 dB averaged at 2 adjacent test frequencies in at least 1 ear
**2**	Threshold shift of greater than 25 dB averaged at 2 adjacent test frequencies in at least 1 ear
**3**	Threshold shift of greater than 25 dB averaged at 3 consecutive test frequencies in at least 1 ear with therapeutic intervention indicated
**4**	Decreased hearing to profound bilateral loss, absolute thresholds above 80 dB HL at 2 kHz and above
